# Multiple QTLs Linked to Agro-Morphological and Physiological Traits Related to Drought Tolerance in Potato

**DOI:** 10.1007/s11105-014-0824-z

**Published:** 2014-11-25

**Authors:** M. Awais Khan, David Saravia, Susan Munive, Flavio Lozano, Evelyn Farfan, Raul Eyzaguirre, Merideth Bonierbale

**Affiliations:** International Potato Center, P.O. Box 1558, Lima 12, Peru

**Keywords:** Drought tolerance, QTL, Potatoes, *Solanum phureja*, Genetic map

## Abstract

Dissection of the genetic architecture of adaptation and abiotic stress-related traits is highly desirable for developing drought-tolerant potatoes and enhancing the resilience of existing cultivars, particularly as agricultural production in rain-fed areas may be reduced by up to 50 % by 2020. The “DMDD” potato progeny was developed at International Potato Center (CIP) by crossing the sequenced double monoploid line DM and a diploid cultivar of the *Solanum tuberosum* diploid *Andigenum Goniocalyx* group. Recently, a high-density integrated genetic map based on single nucleotide polymorphism (SNP), diversity array technology (DArT), simple sequence repeats (SSRs), and amplified fragment length polymorphism (AFLP) markers was also made available for this population. Two trials were conducted, in greenhouse and field, for drought tolerance with two treatments each, well-watered and terminal drought, in which watering was suspended 60 days after planting. The DMDD population was evaluated for agro-morphological and physiological traits before and after initiation of stress, at multiple time points. Two dense parental genetic maps were constructed using published genotypic data, and quantitative trait locus (QTL) analysis identified 45 genomic regions associated with nine traits in well-watered and terminal drought treatments and 26 potentially associated with drought stress. In this study, the strong influence of environmental factors besides water shortage on the expression of traits and QTLs reflects the multigenic control of traits related to drought tolerance. This is the first study to our knowledge in potato identifying QTLs for drought-related traits in field and greenhouse trials, giving new insights into genetic architecture of drought-related traits. Many of the QTLs identified have the potential to be used in potato breeding programs for enhanced drought tolerance.

## Introduction

Water shortage is already one of the main problems faced by agriculture production systems globally and results in huge annual yield losses (Godfray et al. 2010). On top of this, climate change is leading to higher temperatures, reduced water availability, and more frequent and unexpected patterns of drought. Potato is an efficient water user, described as providing more calories per unit of water used than many other agricultural crops but is sensitive to water shortages (Iwama 2008). Drought results in reduced vegetative growth, leaf area, plant height, and tuber yield. The potato crop requires a regular water supply to maintain high quality and production potential, but the specific amount of water needed depends on the developmental stage of the plant, soil type, as well as other climatic factors such as temperature and irradiation. In addition, drought has different levels of impact on quality and overall yield depending on timing, frequency, and duration (Van Loon 1981; Schafleitner et al. 2007). Water shortage has a particularly drastic effect on yield if it occurs at the tuber initiation stage. Response to drought stress is a complex phenomenon that is manifested by several traits that each has small individual contributions, including several agro-morphological features, root system architecture as well as physiological parameters (Heuer and Nadler 1998; Onder et al. 2005; Iwama 2008). The potato germplasm exhibits considerable diversity for drought adaptation traits indicating the potential for improving these traits, ultimately leading to enhanced drought tolerance (Schafleitner et al. 2007; Anithakumari et al. 2012; Cabello et al. 2014).

There have been several studies in crop species focused on understanding the coordination of morphological and physiological traits under different water-limited conditions in order to exploit the genetic variation in these traits in breeding programs (reviewed in detail in Tuberosa and Salvi 2006; Cattivelli et al. 2008; Tester and Langridge 2010). These studies emphasized detailed characterization of the experimental environment, including soil sampling, monitoring weather data during the course of experiments, and well-defined treatments and good control on amount of input water for proper interpretation of results, as described by Reynolds et al. (2009).

There are a large number of tools and methods available to define phenotypic and physiological responses of crops such as wheat to drought for identification of marker-trait associations and gene discovery (Fischer and Maurer 1978; Reynolds et al. 2009; Fleury et al. 2010). The output of several studies has resulted in allowing informed decisions for focusing on important traits and combinations of traits that can lead to climate-smart varieties in these crops (Bruce et al. 2002; Ribaut and Ragot 2007; Edmeades 2013). However, most of these traits are complex and make small contributions to overall phenotype (Blum 2005). Therefore, it is not easy to improve these traits using traditional selection in a breeding program. Molecular markers linked to complex quantitative traits offer time-efficient potential for improving traits with minor effects (Khan and Korban 2012). In upland rice, a large-effect QTL for grain yield for drought stress conditions at reproductive stage was identified (Bernier et al. 2007), and markers associated with this QTL were used to select drought-tolerant donors in upland and lowland-adapted populations and breed drought-tolerant rice, e.g., IR64 (Kumar et al. 2008). These studies not only identified drought tolerance-related traits under controlled and field conditions, architecture and relationship of these traits with each other but also identified the markers linked to them for use in breeding programs (Tuberosa and Salvi 2006; Cattivelli et al. 2008).

In potato, there are several studies that have been conducted to characterize drought tolerance but not many studies to dissect the genetic basis of these traits (reviewed in Monneveux et al. 2013). The availability of potato genome sequence provides a great resource to develop molecular markers and identify QTLs linked to these traits. So far, there have been two studies describing identification of QTLs linked to drought stress tolerance and recovery potential in a greenhouse and polyethylene glycol (PEG)-induced in vitro water-deficit conditions in diploid potato mapping populations (Anithakumari et al. 2011; Anithakumari et al. 2012). A total of 23 QTLs were identified in the in vitro experiment under controlled stress and recovery treatments, explaining from 10.3 to 22.4 % of the variance for phenotypic traits (Anithakumari et al. 2011). The greenhouse experiment identified 47 QTLs, of which 28 were drought-specific, 17 under recovery treatment, and 2 under well-watered conditions (Anithakumari et al. 2012).

A dense genetic and physical map for diploid backcross progeny (DMDD) of potato was recently constructed with a total of 2469 markers, including simple sequence repeats (SSRs), diversity array technology (DArT), and SNPs (Sharma et al. 2013). Using the same genotypic data of these markers for DMDD from Sharma et al. (2013), we constructed maternal and paternal maps to carry out the first QTL study for drought tolerance in potato in greenhouse and field conditions. We evaluate a number of traits to examine their link to drought tolerance and to identify the genomic regions associated to these traits.

## Materials and Methods

### Experimental Design

A BC1 biparental diploid mapping population called DMDD with 180 progeny plants was used in this study. DMDD was developed by crossing a homozygous doubled monoploid of the sequenced *Solanum phureja* “DM” (Potato Genome Sequencing Consortium 2011) with a heterozygous diploid cultivar of the *Solanum tuberosum* diploid *Andigenum Goniocalyx* group “DI,” of which one F1 individual was backcrossed with DI.

The drought tolerance of DMDD progeny was evaluated in two trials in Peru using augmented block design. One trial was conducted in a greenhouse in CIP’s tropical highland station in Huancayo and the second, under field conditions between May and August 2013 in Paucartambo (10° 53' 0" S, 75° 57' 0" W and 2950 masl), a tropical highland location in Peru. Soil samples were collected randomly across 12 locations in the field with 12 subsamples per 0.5 ha along a zigzag pattern through the field using an auger to 15–30 cm and to 30–50 cm tillage depths to test the homogeneity in field. Per sample, 500 g to 1 kg soil was analyzed at the Soil Testing Laboratory at Universidad Nacional Agraria la Molina, Lima, Peru.

Tuber seeds were produced in CIP’s highland station in Huancayo. They were kept in warm storage at CIP for breaking dormancy and tubers with uniform sprouts were planted. The 180 genotypes were randomly assigned to blocks of equal size with 10 plants per genotype and five checks to control for variation between the blocks. Progeny plants of DMDD were used as checks to ensure similar maturity of the checks and the mapping population. The number of checks and blocks for the experiment was defined as error *df* = (*r* − 1)(*c* − 1) = 10, where *c* = number of different checks per block and *r* = number of blocks = number of replicates of a check. Ten (10) is the degrees of freedom (*df*) for error in the ANOVA of checks to ensure reliable estimate of variance components.

Both trials had two water treatments, well-watered (WW) with normal irrigation (every third day) until harvest and terminal drought (TD), where water was withheld after 60 and 62 days after planting (DAP) in greenhouse and field, respectively. The plants in greenhouse experiment were planted in pots, and due to space limitations, treatments were put in adjacent greenhouses. Drip irrigation was installed both in field and greenhouse, and after hilling, a second line was added in the field. Water flow was checked at different places in the field and greenhouse to ensure constant water pressure. Throughout the experiment, a weather station model U30 HOBO (Onset Corporation, Bourne, MA, USA) recorded relative humidity, temperature, water quantity, water pressure deficit, wind velocity, wind direction, precipitation, solar irradiation, and photosynthetically active radiation (PAR) every 15–30 min/day. Additionally, in both the greenhouse and field, HOBO U23 Pro v2 data logger (Onset Corporation, Bourne, MA, USA) recorded temperature, relative humidity, and soil temperature. Tubers were planted manually at 8–10 cm depth in the field with distance between rows at 70–90 cm and between plants 25–30 cm. During the experiment, pest management and weeding were performed in all treatments in the same manner, as required. Fertilization was done manually at the time of tuber planting using organic manure, and hilling was done at around 30 days after planting (same dose and frequency).

### Trait Evaluation

A large number of traits were evaluated in both greenhouse and field trials using a predefined protocol at different developmental stages of the crop, including prestress and post-stress trait evaluations and harvest and postharvest evaluations. The traits were evaluated at similar days after planting for both treatments in both locations with few exceptions. A schedule of evaluations in each trial is provided in Table 1.

**Table 2:** Weather data collected over a growing period in field experiment at Paucartambo and greenhouse in Huancayo in 2013. DMDD population (BC1 progeny) was grown in a field in Paucartambo between May and August 2013 while greenhouse experiment was conducted between March and July 2013 in Huancayo, Peru

Location/treatment	Parameter	March	April	May	June	July	August
HYO/WW	Relative humidity (%)	64.37	66.96	73.43	70.32	62.44	–
	Maximal temperature (°C)	31.51	37.04	34.78	34.28	29.92	–
	Minimal temperature (°C)	5.87	4.17	3.56	1.29	2.18	–
	Mean temperature (°C)	16.44	15.26	14.01	13.23	12.64	–
	Intensity (lum/ft^2^)	–	891.90	928.82	839.90	838.42	–
HYO/TD	Relative humidity (%)	62.26	64.47	70.55	60.76	49.51	–
	Maximal temperature (°C)	32.67	36.15	32.28	35.80	30.29	–
	Minimal temperature (°C)	6.46	4.51	3.72	1.40	2.32	–
	Mean temperature (°C)	17.11	15.86	14.12	14.08	11.56	–
	Intensity (lum/ft^2^)	–	802.80	733.29	676.11	373.50	–
PTBO	Relative humidity (%)	–	–	88.06	88.76	84.49	79.56
	Maximal temperature (°C)	–	–	20.67	21.06	21.20	23.14
	Minimal temperature (°C)	–	–	6.97	4.64	2.90	4.12
	Mean temperature (°C)	–	–	13.46	12.72	11.93	13.12
	Rainfall (mm)	–	–	5.00	55.20	25.80	2.20
	PAR (uE)	–	–	344.90	317.90	367.00	446.94
	Wind speed (km/h)	–	–	1.51	1.28	1.44	1.77
	Gust speed (km/h)	–	–	5.03	4.34	4.87	5.46

*HYO/WW* well-watered greenhouse Huancayo, *HYO/TD* terminal drought greenhouse Huancayo, *PTBO* Paucartambo

**Table 1:** Trait evaluation schedule over a growing period in both field experiment at Paucartambo and greenhouse in Huancayo in 2013. Schedule is shown as days after planting (DAP), trait abbreviations are also shown

Traits evaluated	Abbreviations	Evaluation schedule in days after planting (DAP) in greenhouse (G) and field (F) trial							
		49–50	60–63	70	76–80	84	91–94	98	117
Plant height (cm)	PH	F	G		GF				
Stem diameter (mm)	SD		F		GF	G			
Reflectance-NDVI	NDVI				G	G	F	F	
Chlorophyll content—SPAD	SPAD		G		GF	G			
Biomass fresh weight (g)	BWf								GF
Stems and leaves fresh weight (g)	SLWf								GF
Stems and leaves dry weight (g)	SLWd								G
Biomass dry weight (g)	BWd								G
Tuber number	TN								GF
Tubers fresh weight (g)	TWf								GF
Tubers weight (g) dry	TWd								G
Tuber dry matter content (%)	TDMC								G
Harvest index (g g−1) dry weight	HId								G
Harvest index (g g−1) fresh weight	HIf								GF

G, F, and GF represent data taken in greenhouse only, field only, or both in greenhouse and field, respectively

#### Morphological and Physiological Traits

The plant height (cm) of the main stem of each clone was measured from the tip of the plant to the ground level using a ruler. Stem diameter (cm) was also measured just above the first leaves using Vernier calipers. Chlorophyll content in the plant leaf was taken from three (3) leaflets/plant/clone, from the third fully developed mature leaf using a chlorophyll meter (SPAD-502, Minolta). Normalized difference vegetation index (NDVI) was determined by using a FieldScout CM1000 sensor which measures light reflected by the crop in the red and near infrared spectral bands in the field.

#### Evaluations at Harvest

Plants were extracted from soil with a shovel, with the same depth and distance from each plant maintained to ensure consistent extraction of the root system. The number of tubers/plant (N pta-1) for each plant was counted in all treatments at harvest, counting only tubers with at least twice the diameter of the stolon. The total weight (g) of fresh biomass (g), stems and leaves fresh weight (g), and total fresh weight (g) of tubers for each plant were measured using an analytical balance for each treatment and genotype.

#### Postharvest Evaluations

Total biomass, stems and leaves, and roots of each plant from both treatments from the greenhouse experiment in Huancayo were weighed before and after oven-drying for 2 days at 80 °C to measure dry weight (g). Tubers of each plant from all treatments were cut, and 200 g fresh weight samples were put in paper bags for oven-drying for 4 days at 80 °C and then weighed on an analytical balance for estimating tuber dry weight (g). Tuber dry matter content (%) was calculated by dividing the tuber dry weight by total fresh tuber weight used. Afterwards, the value of dry matter content (%) was multiplied by total fresh tuber weight (g) to estimate total tuber dry weight (g).

In the field experiment in Paucartambo, because of logistical issues, only tuber fresh weight (g) was taken as described above. We also calculated total dry biomass weight (g) by adding dry weight (g) of leaves and stems, tubers, and roots of the corresponding plants in the greenhouse experiment in Huancayo. For harvest index (HI) based on fresh harvest (g), total fresh tuber weight (g) was divided by total fresh biomass (g) of the corresponding plant, and HI on dry material was calculated as estimated tuber dry weight (g) divided by total biomass on dry weight basis (g).

## Statistical Analysis

Trait evaluation data from both trials were searched for outliers. Trait distribution, mean, the range of each trait and broad-sense heritability at treatment level in both trials as well as % reduction in each individual experiment for each trait was calculated. The % reduction was calculated by dividing the trait mean in WW with the corresponding trait mean from TD treatment and multiplying by 100 as (100 − (mean at WW / mean at TD × 100)).

Checks in the augmented design were used to adjust for variation between the blocks, and the mean of each genotype was calculated after correcting for the variation due to the position in the greenhouse in Huancayo or field location in Paucartambo. This adjusted mean was used to estimate the trait heritability using R. Pearson correlation was performed to investigate the relationship of the traits at the treatment and experiment levels. Heatmap.2 package was used to draw the heat map based on trait correlations analysis in R.

### Genetic Map Construction and QTL Analysis

The marker data and genetic map order and positions developed by Sharma et al. (2013) were used to construct the genetic map developed in this study. JoinMap version 4 (Van Ooijen 2006) was used to construct male and female genetic maps. The marker data that had more than two alleles was split and assigned accordingly to the respective source parent. Default options with Kosambi function and “fixed order” option in JoinMap based on marker orders of map published by Sharma et al. (2013) were used to construct both parental maps. Adjusted mean values of the 60 traits measured on the 180 progeny genotypes in both environments, five traits specific to Paucartambo and one to Huancayo, and the two parental maps were used separately for QTL mapping. The data for both treatments, well-watered and terminal drought, were used separately for QTL analysis, within locations; and QTLs detected for one treatment, but not both, were taken to be stress related. QTL analysis was performed using Kruskal-Wallis test and interval mapping in MapQTL 5 (Van Ooijen and Kyazma 2009) with default options. Permutation test was performed to find the threshold for declaring a QTL significant in MapQTL 5. Additionally, a confidence interval for likelihood position of QTL was estimated at 1 and 2-LOD score drop on both sides of each QTL peak.

## Results

### Experimental Conditions and Irrigation Regimes

The relative humidity ranged from 62 to 73 % in WW in the greenhouse with 67 % at tuber initiation stage and 49 to 70 % in TD treatment, being 64 % at tuber initiation stage. The radiation intensity consistently declined in TD treatment while staying almost constant over the entire growing period in WW (Table 2). Relative humidity, maximum, minimum, mean temperature as well as radiation intensity were similar in both treatments in greenhouses in Huancayo for the first 2 months (March and April). However, we saw big differences in radiation intensity for 3 months (May–July), being significantly higher in WW greenhouse (Table 2). Also, relative humidity in normal treatment in June and July was significantly lower in TD greenhouse (Table 2). The relative humidity was considerably higher in Paucartambo, ranging from 79 to 88 % between May and August, and consistently decreased with increase in temperature, ranging from 20 to 23 °C over the growing period. The temperature was 21 °C at tuber initiation stage in Paucartambo. Minimum temperature was similar at tuber initiation stage in the greenhouse; however, there were wider fluctuations between minimum and maximum temperatures in Paucartambo compared to the greenhouse experiment. Also, there was a wide range of rainfall, from 2 to 55 mm, with heavy rain in June (55.2 mm) and July (25.8 mm) and photosynthetically active radiation (PAR) ranged from 317 to 446 uE.

### Relationship of Traits, Trials, and Irrigation Regimes

The data for five plants per genotype were adjusted using the checks present in each block of the augmented block design and used to obtain average of each genotype. Frequency distribution (data not shown) showed that a majority of the trait data from both trials and treatments was normally distributed.

**Fig. 1:**
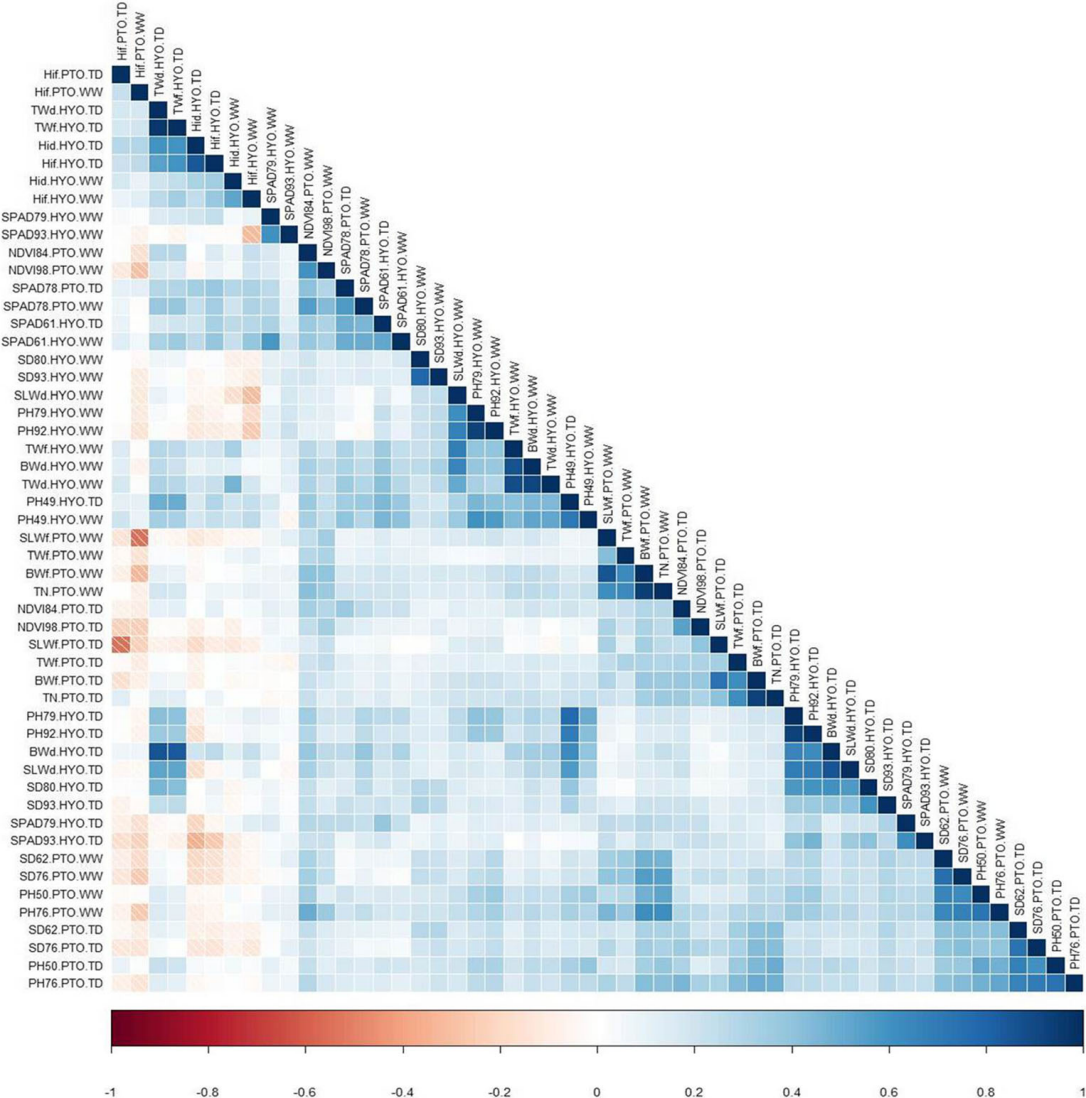
Relationships among agro-morphological traits evaluated in well-watered and terminal drought treatments based on Pearson correlation in greenhouse trial in Huancayo and field trial in Paucartambo. Correlation values vary from 1 (highly positively correlated) to 0 (no correlation) and −1 (highly negatively correlated)

Biomass and tuber weight (g) had the highest reduction between WW and TD treatments across all genotypes in field and greenhouse trials, with a range from 43.4 to 59.4 %, meaning that only half of the biomass and tuber weight (g) was produced in TD. Maximum TWd in the greenhouse at Huancayo was 66.8 g/plant compared to 30.9 g/plant in WW. The reduction in TN was 36.9 %, with a maximum of 131.3 tubers/plant in WW compared to 84.3 tubers/plant in TD (Table 3). In the field at Paucartambo, there is no obvious difference in plant height (PH) in both water regimes, but in greenhouse on average, plants were 21 to 28 % taller in TD treatment. The heritability of traits varied from 21.2 to 92.3, generally TD in Paucartambo showed lower heritability values. Most traits measured show high to moderate heritability, with some exceptions. HIf showed the lowest heritability (25–32.3) in Paucartambo in both treatments. In TD, SLWd in the greenhouse increased by 33.7 %, similar to the trend seen in PH in the greenhouse (Table 3). HI calculated based on fresh and dry weights in both trials and treatments; group together and are positively correlated with each other. In the field, within both treatments, PH is positively correlated with SD. Between treatments; PH is also positively correlated with SD, though to a lesser degree. NDVI showed a significant correlation between PH in both treatments in Paucartambo. NDVI 84 and 98DAP in Paucartambo WW were negatively correlated with HI from fresh weight in same location and treatment, NDVI98 with stronger negative correlation (Fig. 1). SPAD 93DAP in Huancayo WW was negatively correlated with HIf in same location and treatment. SPAD79 and 93 in Huancayo TD were negatively correlated with HIf and HId for Huancayo TD, as well as HIf in Paucartambo WW and TD. Generally, traits show correlations and tend to cluster together within trials and treatments, not across them (Fig. 1).

**Table 3:** Agro-morphological and physiological traits that were evaluated in “DMDD” mapping population in field experiment at Paucartambo (Ptbo), Peru, and greenhouse in Huancayo (Hyo), Peru, in 2013

Variable	Abbreviations	Time/DAP	Trial	Treatment	Mean	Range	h2	% reduction
Biomass	BWd	At harvest	Hyo	WW	52.3	12.7–97.4	76.2	43.4
				TD	29.6	2.6–56.6	92.3	
			Ptbo	WW	727.4	21.7–2767	82.7	59.4
				TD	295.2	41.7–966.7	52.5	
Harvest index	HId	At harvest	Hyo	WW	0.6	0.3–0.8	57.7	16.7
				TD	0.5	0.1–0.7	70.9	
	HIf			WW	0.6	0.2–0.8	56.3	0
				TD	0.6	0.1–0.9	62.9	
			Ptbo	WW	0.8	0.4–1	32.3	0
				TD	0.8	0.4–1	25	
NDVI	NDVI	84	Ptbo	WW	0.8	0.5–0.9	73.7	12.5
		84		TD	0.7	0.5–0.8	21.2	
		98		WW	0.8	0.6–0.9	60.8	12.5
		98		TD	0.7	0.4–0.8	43.8	
Plant height	PH	49	Hyo	WW	56.1	15.3–83.3	87.9	−23.5
		49		TD	69.3	16.3–107.2	90.7	
		79		WW	78.5	42.8–125.5	71.4	−28.3
		79		TD	100.7	38.5–151.2	76.9	
		92		WW	79.3	47.8–134.2	75.3	−21.3
		92		TD	96.2	38.8–144	59.1	
		50	Ptbo	WW	31.2	9.7–55.7	64.1	3.2
		50		TD	30.2	6–58	69.1	
		76		WW	42.2	9–76	74.1	5.9
		76		TD	39.7	6–66.7	66.5	
SPAD	SPAD	61	Hyo	WW	38.6	21.3–48.9	69.6	5.2
		61		TD	36.6	16.2–48.4	62.4	
		79		WW	33.7	23.4–45.2	44.1	−15.4
		79		TD	38.9	15.3–53.3	57	
		93		WW	28.4	11.5–41.4	35.1	−20.4
		93		TD	34.2	8.3–51.6	46.3	
		78	Ptbo	WW	37.3	18.5–47	66.9	−4.8
		78		TD	39.1	21.5–47.7	73.8	
Stem diameter	SD	80	Hyo Ptbo	WW	6.9	4.6–9.3	60.1	7.2
		80		TD	6.4	3.1–9.7	69.9	
		93		WW	7	4.2–9.4	77.6	22.9
		93		TD	5.4	2.7–8	68.5	
		62		WW	6.4	3–10.1	48.4	−1.6
		62		TD	6.5	2.7–10.8	66.4	
		76		WW	6.8	3.3–10	72.2	14.7
		76		TD	5.8	2.9–10.1	62.5	
Stem leaf weight	SLWd	At harvest	Hyo	WW	8.3	2.9–28.2	85.6	−33.7
				TD	11.1	1–28.3	83.4	
	SLWf		Ptbo	WW	226.1	3–1292	72.4	62.7
				TD	84.4	6.5–461.7	49.6	
Tuber number	TN	At harvest	Ptbo	WW	31.9	2.3–131.3	79.3	36.9
				TD	20.1	2.3–84.3	71.1	
Tuber weight	TWd	At harvest	Hyo	WW	33.9	6.3–66.8	74.4	59
				TD	13.9	0.3–30.9	87.3	
	TWf			WW	121.4	27.3–266	69.9	53.1
				TD	56.9	1.8–131.2	90.3	
			Ptbo	WW	520	13.3–1677	75.1	56.2
				TD	228	30–633.3	55.6	

Abbreviations of variables, timing of evaluations, mean, range, narrow sense heritability (h2), and % reduction of traits for WW and TD in Hyo and Ptbo are shown

### Genetic Maps

We established 12 linkage groups corresponding to the genetic maps published in Sharma et al. (2013), one for each parent. The maternal map had 424 markers with 35 markers per chromosome (CHR) on average while the paternal map had 344 markers with 29 markers per CHR on average. Both female and male maps had similar lengths of 785 and 704.4 cM, with an average of 65.4 and 58.7 cM per CHR, respectively. Average intervals between markers on each CHR were 1.9 and 2.1 cM for maternal and paternal maps, respectively. The maximum interval between markers was 24.7 cM on CHR 3 in maternal map while 21.5 cM on CHR 11 was the longest interval between the markers in paternal map (Table 4).

**Table 4:** Key features including number of markers, maximum length, average and maximum marker interval per chromosome (CHR) of parental maps based on a BC1 biparental diploid mapping population called “DMDD” with 180 progeny plants

Chromosome (CHR)	No. of markers	Max. length of LG (cM)	Av. marker interval (cM)	Max. marker interval (cM)				
	M	P	M	P	M	P	M	P
01	66	35	85.1	57.7	1.3	1.7	15.5	12.8
02	39	46	63.1	64.3	1.7	1.4	12.5	21.4
03	52	26	87.4	59.9	1.7	2.4	24.7	16.5
04	30	26	85.1	73.6	2.9	2.9	19.8	15.8
05	10	14	54.8	39.9	6.1	3.1	12.7	14.2
06	39	25	63.3	57.6	1.7	2.4	7.2	9.9
07	29	31	66.8	60.5	2.4	2.0	14.2	18.2
08	34	25	55.3	54.1	1.7	2.3	11.1	17.2
09	28	45	34.2	72.3	1.3	1.6	8.4	12.8
10	53	23	65.0	62.3	1.2	2.8	9.9	21.5
11	29	26	57.3	43.2	2.0	1.7	10.5	7.4
12	15	22	67.5	59.0	4.8	2.8	23.0	10.6
Total	424	344	785	704.4				
Average	35.3	29	65.4	58.7	1.9	2.1		

These paternal (P) and maternal (M) maps are based on genotyping data of SSRs, DArT, and SNPs (Sharma et al. 2013)

### QTL Mapping

**Fig. 2:**
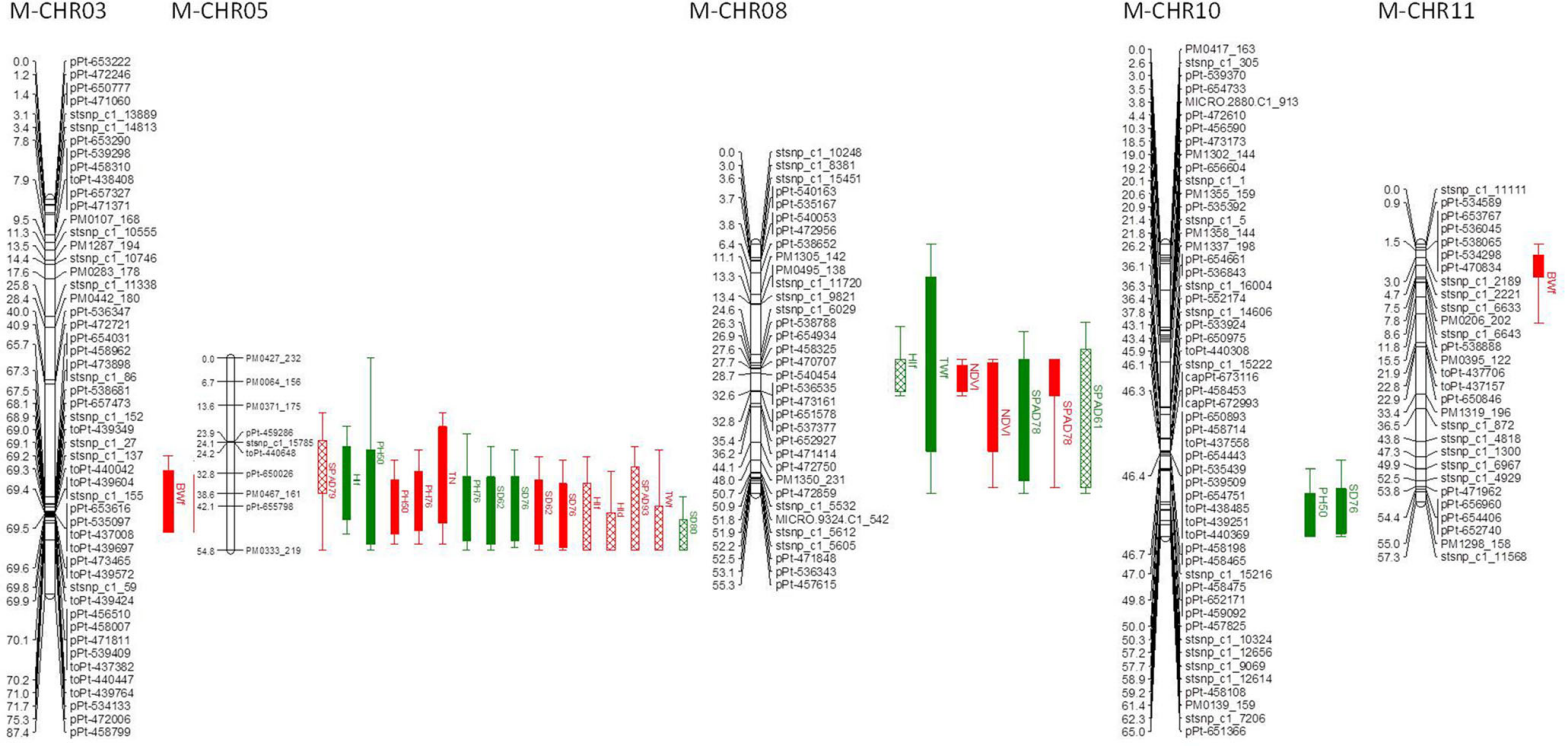
QTLs identified on maternal map of BC1 biparental diploid mapping population “DMDD” in drought trials. For each QTL, trait name and abbreviations (Table 1), trial and the 2-LOD support interval for likelihood of the position are provided. The *bar* indicates the 2-LOD support interval of QTL whereas QTLs in terminal drought and well-watered treatments are *red* and *green*, respectively. QTLs identified in greenhouse trial in Huancayo are represented by *crisscross fill* while QTLs identified in Paucartambo are represented by *full color*

**Fig. 3:**
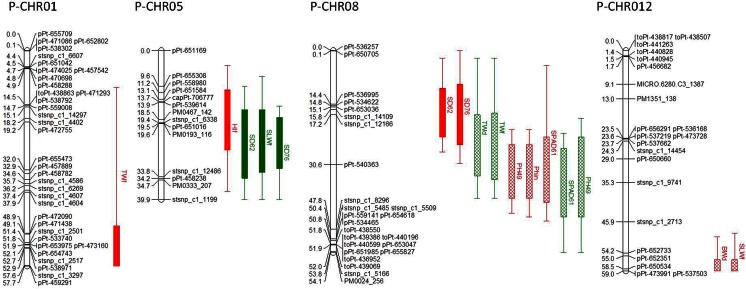
QTLs identified on paternal map of BC1 biparental diploid mapping population “DMDD” in drought trials. For each QTL, trait name and abbreviations (Table 1), trial and the 2-LOD support interval for likelihood of the position are provided. The *bar* indicates the 2-LOD support interval of QTL whereas QTLs in terminal drought and well-watered treatments are *red* and *green*, respectively. QTLs identified in greenhouse trial in Huancayo are represented by *crisscross fill* while QTLs identified in Paucartambo are represented by *full color*

Based on results from the permutation test, a 3.5 logarithm of odds (LOD) score was declared as QTL significant threshold at 99 % confidence. A total of 43 QTLs associated with multiple agro-morphological and physiological traits were identified in both parental maps. A total of 29 QTLs were identified on the maternal map for eight traits. Of these QTLs, 8 were identified in Huancayo greenhouse experiment while 21 QTLs were identified in the field experiment conducted in Paucartambo (Fig. 2; Table 5). On the paternal map, there were a total of 16 QTLs identified for seven traits, 9 in the greenhouse in Huancayo, and 7 in field trial in Paucartambo (Fig. 3; Table 6). No QTL for NDVI or tuber number were identified in the paternal map compared to the maternal map. The QTLs identified for the maternal map are on CHR 3, 5, 8, and 10 and for paternal map are on CHR 5, 8, and 12. The LOD scores of significant QTLs ranged from 3.51 to 8.63 and from 3.56 to 5.66 for maternal and paternal maps. Similarly, the range of phenotypic variation explained ranged from 9.7 to 23.0 and from 10.0 to 16.8 % for maternal and paternal maps, respectively. The highest number of QTLs was detected on CHR 5, with 16 on the maternal map, followed by 9 QTLs on CHR 8 on the paternal and 7 QTLs on CHR 8 of the maternal parent.

**Table 5:** QTL related to agro-morphological and physiological traits on maternal map of BC1 biparental diploid mapping population “DMDD.” For each QTL, trait name, trial, treatment, timing of trait evaluation, chromosome (CHR), peak position, LOD, and locus at peak are provided

Trait	DAP and additional features	Trial	Treatment	CHR	Peak position	Locus at peak	LOD	PVE	Interval (2-LOD)
Biomass	Fresh	Ptbo	TD	3	68.088	pPt-657473	6.88	20.2	55.9–81.3
	Fresh	Ptbo	TD	11	4.69	stsnp_c1_2221	3.35	10.6	0–17.5
Harvest index	Fresh	Ptbo	WW	5	34.808		6.43	18.4	19.6–50.1
	Fresh	Hyo	TD	5	48.069		3.71	13.6	28.2–54.8
	Dry	Hyo	TD	5	49.069		7.22	24.6	32.2–54.8
	Fresh	Hyo	WW	8	26.272	pPt-538788	5.01	15	18.4–33.8
NDVI	84	Ptbo	TD	8	28.675		6.39	17.5	25.6–33.8
	98	Ptbo	TD	8	32.567	pPt-536535	4.05	11.2	25.6–54.1
Plant height	50	Ptbo	TD	3	69.384	toPt-439604	4.46	12.1	56.9–86.3
	50	Ptbo	WW	5	38.634	PM0467_161	3.52	9.7	0–54.8
	50	Ptbo	TD	5	39.634		6.01	16.7	29.2–53.1
	76	Ptbo	TD	5	39.634		6.62	18.5	26.2–53.1
	76	Ptbo	WW	5	45.069		4.66	14.4	21.6–54.8
	50	Ptbo	WW	10	58.919	stsnp_c1_12614	3.63	9.9	49.8–65
SPAD	79	Hyo	TD	5	32.808	pPt-650026	3.93	12	15.6–54.8
	93	Hyo	TD	5	49.069		5.48	18.4	25.2-54.8
	78	Ptbo	WW	8	32.567	pPt-536535	4.23	11.5	19.4–55.3
	78	Ptbo	TD	8	32.567	pPt-536535	4.87	13.4	25.6–54.1
	61	Hyo	WW	8	50.726	pPt-472859	3.87	11.8	17.4–55.3
Stem diameter	62	Ptbo	WW	5	46.069		5.13	15.7	25.2–54.8
	76	Ptbo	WW	5	46.069		6.76	20.3	26.2–54.1
	62	Ptbo	TD	5	47.069		5.17	16.8	28.2–54.8
	76	Ptbo	TD	5	47.069		5.23	17	29.2–54.8
	80	Hyo	WW	5	54.818	PM0333_219	3.51	10.8	39.6–54.8
	76	Ptbo	WW	10	62.297	stsnp_c1_7206	3.59	9.8	48–65
Tuber number	117	Ptbo	TD	5	42.069	pPt-655798	3.83	10.9	15.6–53.1
Tuber weight	Fresh	Ptbo	TD	3	69.316	toPt-440042	7.51	21.9	56.9–82.3
	Fresh	Hyo	TD	5	51.069		3.73	12.8	26.2–54.8
	Fresh	Ptbo	WW	8	26.272	pPt-538788	3.56	9.9	0–55.3

Moreover, the 2-LOD support interval for likelihood of the position of QTL and phenotypic variation explained (PVE) of each QTL is provided

**Table 6:** QTLs related to agro-morphological and physiological traits on paternal map of BC1 biparental diploid mapping population “DMDD.” For each QTL, corresponding trait name, trial, treatment, timing of trait evaluation, chromosome (CHR), peak position, logarithm of odds (LOD), and locus at peak are provided. Moreover, the 2-LOD support interval for likelihood of the position of QTL and phenotypic variation explained (PVE) of each QTL is provided

Trait	DAP and additional features	Trial	Treatment	CHR	Peak position	Locus at peak	LOD	PVE	Interval (2-LOD)
Biomass	Dry	Hyo	TD	12	57.984		4.09	13.4	49.9–59
Harvest index	Fresh	Ptbo	TD	5	17.872		3.56	10.4	4-37.7
Plant height	49	Hyo	TD	8	30.584	pPt-540363	3.92	12	4.1–45.6
	Increase^1^	Hyo	TD	8	30.584	pPt-540363	5.66	16.8	21.2–43.6
	49	Hyo	WW	8	34.584		3.69	13.3	18.2–54.1
SPAD	61	Hyo	TD	8	30.584	pPt-540363	4.2	12.8	21.2–44.6
	61	Hyo	WW	8	33.584		4.71	15.4	22.2–54.1
Stem diameter	62	Ptbo	WW	5	23.559		3.87	12.4	9.6–39.9
	76	Ptbo	WW	5	24.559		4.99	16.5	14.9–39.9
	62	Ptbo	TD	8	17.229	stsnp_c1_12166	3.86	10.7	2.1–27.2
	76	Ptbo	TD	8	17.229	stsnp_c1_12166	3.56	10	0–30.2
Stem leaf weight	Fresh	Ptbo	WW	5	23.559		3.7	12.3	7–39.9
	Dry	Hyo	TD	12	57.984		3.98	13	48.9–59
Tuber weight	Dry	Hyo	WW	8	25.229		4.1	14.6	2.1–39.6
	Fresh	Hyo	WW	8	25.229		3.71	12.9	2.1–39.6
	Fresh	Ptbo	TD	1	51.39	Stsnp_c1_2501	3.01	9.5	9.9–57.7

^1^Plant height increase is the growth rate calculated as PHI PH DAP79–PH DAP49

### Potentially Drought-Specific QTLs

QTLs identified in either of the treatments but not the other within a trial were taken as potentially drought-specific. Overall, comparing the treatments, 24 QTLs were identified in TD treatment and 19 in WW treatment in both trials. In the maternal map, Paucartambo and Huancayo had 9 and 7 QTLs, respectively, specific to drought treatment (Fig. 2; Table 5), of which 5 QTLs identified in TD in Huancayo and 2 QTLs were identified in WW, while in Paucartumbo, 6 QTLs were identified in TD and 3 in WW. The QTLs identified for TD in maternal parent were on CHR 3, 5, 8, and 11 and for WW on CHR 5, 8, and 10. The strongest QTL for the maternal map was identified in CHR 3 at 69.316 cM for TWf in Paucartambo for TD treatment, with a LOD score of 7.51, and explaining 21.9 % of phenotypic variation. A strong QTL with LOD score of 6.88 and phenotypic variation explained (PVE) of 20.2 % was also identified at the same position of the maternal map for BWf in Paucartambo as well. The QTL for HId was the strongest on CHR 5 with a LOD score of 7.22 and PVE of 24.6 %. Another QTL for HIf with LOD of 6.43 and PVE of 18.4 % were also identified on CHR 5 at a similar position in both trials. Another drought-specific QTL for NDVI was identified in Paucartambo in TD treatment at 84 DAP with the LOD score 6.39 and PVE of 17.5 % and was also identified in the similar map position at 98 DAP, in the maternal map.

In the paternal map, 10 drought-specific QTLs were identified, 5 in Huancayo, and 5 in Paucartambo; and 6 in TD treatment and 4 for WW. QTLs in TD were on CHR 1, 5, 8, and 12 while in WW, QTLs were identified only on CHR 5 and 8. Two QTLs were identified on CHR 12 at 57.9 cM position of the paternal map for biomass and stem leaf weight (a related trait). The QTLs were only identified for TD treatment in Huancayo and had an LOD score of 3.98–4.1 with PVE of ~13 %. A QTL identified in the paternal map for PH increase (increase in height from 49 to 79 DAP) in Huancayo at 30.6 cM for TD was the strongest QTL with LOD score of 5.66 and PVE of 16 % peak at pPt-540363. Additionally, 2 QTLs linked to each TWd and TWf were identified on CHR 8 with LOD score ranging from 3.71 to 4.1 and PVE from 12.9 to 14.6 % at 25.2 cM in WW treatment only (Fig. 3; Table 6).

### Other Significant QTLs

On the maternal map, there are 13 QTLs identified that are not considered drought-specific, either because they are from data taken before the onset of stress or because they were identified in both treatments. For PH, SPAD, and SD, there are 10 QTLs each found in both treatments and on different time points but at the same map position, making 3 QTLs. On the maternal map, QTLs for both SD and PH were identified on CHR 5 with a position of 38–45 cM for PH and LOD score ranged from 3.52 to 6.64, and a position of 46–47 cM and LOD score 5.13–6.76 for SD.

On the paternal map, 6 QTLs are not drought-specific, with 5 on CHR 8 and 1 on CHR 5. The QTLs for SPAD and PH co-localize on CHR 8 at 30-34 cM, with LOD scores ranging from 4.2 to 4.71 for SPAD and from 3.69 to 3.92 for PH, all found in Huancayo. In Paucartambo, 2 QTLs were identified for SD at 62 DAP, in the WW treatment on CHR 5 at 24 cM and with a LOD score of 3.87, and in the TD treatment at CHR 8, 17 cM and a LOD score of 3.86.

## Discussion

A total of 45 QTLs were identified for several agro-morphological and physiological traits in greenhouse and field trials in both well-watered (WW) and terminal drought (TD) treatments. The presence of a large genetic diversity and the segregation of several of these traits led to the identification of this large number of QTLs, suggesting that DMDD is suitable for identification of marker-trait association for adaptation and abiotic stress-related traits, such as drought. The heritability of most of the traits is moderate to high in this study and was found to be quite similar as previously reported (Anithakumari et al. 2011). High heritability and genetic variance are important for predicting the response to selection in crop improvement programs, while low heritability of traits leads to expensive and slow improvement through selection (Piepho and Möhring 2007; Collard and Mackill 2008). QTLs linked to highly heritable traits are also stable across environments (Messmer et al. 2009).

Many of the QTLs are environment specific, found in only one treatment of one trial, indicating the complexity of the traits related to drought tolerance. The expression of a trait and its underlying genetic mechanism could be different because of not only water availability but also the overall environment, including temperature, the local microbiome, soil composition, light intensity, etc. (Tuberosa 2012). In our study, the range of temperatures during the growing season varied between the greenhouse trial in Huancayo and the field trial in Paucartambo (Table 2). Besides other factors such as elevation and day-length, this might have contributed to finding different QTLs in each trial. In Paucartambo, several QTLs were also found consistently in both treatments, indicating that these are not drought-specific QTLs. As WW and TD treatments were in close proximity, it is unlikely that other environmental factors were different. However, in Huancayo greenhouses, we do see differences in additional environmental factors, besides water suspension, between the two treatments. Relative humidity and light intensity decline over time in the TD treatment, starting from the initiation of drought at 60 DAP in May. The complete suspension of watering must have led to low relative humidity measurements in the air (Table 2). This difference in relative humidity might have resulted in a difference in interception of radiation that could be the reason for difference in radiation intensity, especially as it consistently declines in a similar way as the humidity. Therefore, the QTLs found in one treatment but not the other could be due to these environmental differences and not necessarily drought specifically. In addition, low correlation between each trait in different trials and treatments also suggests the multigenic control of the traits and the extent of environmental influence on the expression of QTLs, as has been suggested for other crops as well, such as the common bean (Asfaw et al. 2012). These complex traits are generally controlled not only by many genes individually but also by the interaction of multiple gene pathways and environments, and this complexity is multiplied with the addition of various levels of stress and the stage of the crop cycle (Mathews et al. 2008).

A majority of the QTLs for the agro-morphological traits evaluated in both maternal and paternal maps were found in CHR 5 and 8, including some of the strongest drought-specific ones on CHR 5. In potato, CHR 5 and 8 are hot spots for vigor, maturity, and biomass traits. Several studies have identified QTLs for vigor, maturity, tuber, and biomass on CHR 5 (Schäfer-Pregl et al. 1998; Collins et al. 1999; Oberhagemann et al. 1999; Menéndez et al. 2002; Visker et al. 2003; Ballvora et al. 2007; Danan et al. 2011). QTLs for resistance to pathogens have also been found co-localized at the same position on CHR 5. It is believed that the underlying genes are the same and have pleiotropic effect on many traits (Visker et al. 2003). On CHR 8, Danan et al. (2011) also identified maturity meta-QTLs using a consensus map of potato.

In the maternal parent, 16 QTLs were drought specific, that is, they are found in only one treatment. Messmer et al. (2009) found that QTLs that were stable across years and locations were not stable across water treatments, indicating variable genetic control and activation of different gene pathways for some traits under drought or well-watered environments. The novel QTL identified for fresh tuber weight on CHR 3 at 69.3 cM was the strongest drought-specific QTL, explained 21.9 % of variation, and was found only in Paucartambo TD treatment with low to moderate heritability. This is aligned with previous findings in Andigenum cultivar group and indicates that selection for increased tuber yield under drought conditions may be slow due to its low heritability (Cabello et al. 2014). This QTL might be important for certain environments and offer potential to enhance tuber yield through marker-assisted breeding. Bradshaw et al. (2008) also mapped several QTLs linked to yield, agronomic and quality traits in tetraploid potato; however, due to lack of common markers across both maps, it is difficult to establish corresponding position of QTLs for comparison between the studies. The QTL found on CHR 5 for harvest index, fresh and dry, in Paucartambo and Huancayo, and representing 14 to 25 % of variation, presents an interesting case. In both trials, it is found in only one treatment, which confirms it as a drought-related QTL. As mentioned above, confounding environmental factors that vary between treatments in Huancayo make it less reliable for identifying any QTL as drought specific, but as this QTL was also found in only one treatment in Paucartambo, it is a good candidate despite the fact that in Paucartambo, it is present in the WW while in Huancayo, in the TD treatment. We cannot know with certainty how a QTL interacts with the environment in each specific case; however, future experiments involving multi-environmental trials might be able to further dissect this interaction. A QTL for NDVI was found in Paucartambo in the TD treatment on CHR 8, explaining 17.5 % of variation. NDVI compares the reflectance in near infrared and red wavebands of light, indicating photosynthetic activity and the general health of the plant and is considered a good measure of the drought status of vegetation. Thus, secondary traits such as NDVI are often used as indicator traits for drought resistance and can be used as selection criteria in breeding programs (Yue et al. 2006).

Ten putative drought-specific QTLs were found in the paternal map. Of these, the strongest is in CHR 5 at 24.5 cM, for stem diameter in Paucartambo in the WW treatment, explaining 16.5 % of variation. Some of these QTLs could be redundant, for example, the stem diameter QTL and stem leaf weight QTL identified in Paucartambo on CHR 5 are at similar positions (stem diameter 23–24.5 cM and stem leaf weight fresh 23.5 cM), and as the traits are positively correlated with each other, this region seems to be associated with both traits. Stem diameter can be an important indicator of water status of a plant in different stresses and often responds quickly to water stress by regulation of stomatal opening. Over the longer term, a reduced stem diameter could be indicative of reduced secondary growth (Ohashi et al. 2006). In the greenhouse trial, we find 7 QTLs on CHR 8 for plant height, SPAD, and tuber weight. These 7 are likely 3 QTLs, one each for plant height, SPAD, and tuber weight. The QTL linked to tuber weight was only identified in the WW treatment, for both dry and fresh weight, so it is likely to be drought-linked and is correlated with dry matter content of the tubers. The QTLs for plant height and SPAD are not related to water shortage, as they were identified before the onset of stress treatment or in both treatments.

Measuring many of the same traits as the present study, besides some root and tuber traits, SPAD, and NDVI, Anithakumari et al. (2011) identified 10 QTLs on CHR 2 of potato with in vitro PEG treatments, whereas we did not identify any QTLs on CHR 2. Both studies found QTLs on seven linkage groups, with similarity for CHR 3, 5, 8, and 12. A QTL associated with plant height on CHR 3 explaining 19.3 % of variation may be the same in the present study before stress initiation. Another QTL identified in our study for biomass and stem leaf weight on CHR 12 might be the same QTL found by Anithakumari et al. (2011) for stem fresh weight and stem dry weight. Without common markers on the maps, it is not possible to say with certainty. Surprisingly, Anithakumari et al. (2011) found only 1 QTL on CHR 5, for biomass, while we found a total of 20 from both parental maps, with none for biomass, although some are for related traits such as stem diameter and plant height. In a greenhouse experiment using the same population, Anithakumari et al. (2012) found a majority of maturity-related QTLs on CHR 5, for example, a QTL for plant height was found over two trials as well as a QTL for shoot fresh and dry weight, which might be the same as ours discussed earlier. The study also reported QTLs for a range of developmental traits, with some surprisingly explaining up to 66 % of variation, e.g., for tuber weight. This may be an overestimation stemming from the small population size of 96 individuals. The different QTLs in the present study and in the studies of Anithakumari et al. (2011 and 2012) are likely due to the different populations (CxE, where C is a hybrid between *Solanum phureja* and *Solanum tuberosum* di-haploid), as well as environmental differences such as temperature. The PEG treatment and tissue culture itself (Anithakumari 2011) are also stresses and could therefore identify different QTLs. Moreover, where there are not many common meiotic events between the parents of a cross and where partially informative markers are used to integrate maps can lead to overestimation of effects and errors in marker order (Khan et al. 2012). The interval between markers in our study is less than 2 cM, which ensures identification of small effect QTLs (Table 4).

The majority of the drought-related QTLs reported here explain small to moderate amounts of phenotypic variation, as would be expected from a complex quantitative trait like drought tolerance. This is the first study to our knowledge that identified QTLs for drought-related traits in field and greenhouse trials for potato. It gives insight into genetic architecture of drought-related traits, and the identified QTLs will lead to marker development that can be used in breeding programs for enhanced drought tolerance. Further trials for testing these QTLs in different stress scenarios, including drought initiation at different developmental stages of potato, with varying levels of stress from moderate to recovery in additional environments will expand the knowledge on their role and stability. Afterwards, stable QTLs could be combined by introgressing several small-effect QTLs into an elite background using marker-assisted selection that could lead to development of cultivars with enhanced drought stress tolerance.
